# 4-Hexylresorcinol Loaded Solid Lipid Nanoparticles for Enhancing Anticancer Activity

**DOI:** 10.3390/ph17101296

**Published:** 2024-09-29

**Authors:** Sooho Yeo, Sukkyun Jung, Haneul Kim, Jun-Hyun Ahn, Sung-Joo Hwang

**Affiliations:** 1 Yonsei Institute of Pharmaceutical Sciences, College of Pharmacy, Yonsei University, Incheon 21983, Republic of Korea; 1543sky@naver.com; 2 Research Center of Barunbarum Co., Seoul 06776, Republic of Korea; jsk0314@barunbarum.com; 3 Department of Biopharmaceutical Engineering, Hannam University, 1646 Yuseongdae-ro, Yuseong-gu, Daejeon 34054, Republic of Korea; ajh@hnu.kr

**Keywords:** 4-hexylresorcinol, chemotherapy, solid lipid nanoparticle, anti-cancer therapy, enhanced permeation and retention effect

## Abstract

Background: Cancer is one of the most significant threats to human health. Following surgical excision, chemotherapy is an effective strategy against remaining cancer cells. 4-hexylresorcinol (4-HR) has anti-cancer properties and exhibits hydrophobicity-induced aggregation in the blood that has trouble with targeted tumor delivery and cellular uptake of the drug. The purpose of this study is to encapsulate 4-HR into solid lipid nanoparticles (SLNs) to enhance its anti-cancer effect by avoiding aggregation and facilitating cellular uptake. Methods: 4-HR SLNs were prepared via hot melt homogenization with sonication. SLN characteristics were assessed by analyzing particle size, zeta potential, and drug release. Cytotoxicity, as an indicator of the anti-cancer effect, was evaluated against HeLa (cervical cancer in humans), A549 (lung cancer in humans), and CT-26 (colon carcinoma in mice) cell lines. Results: Particle size ranged from 169.4 to 644.8 nm, and zeta potential ranged from −19.8 to −40.3 mV, which are conducive to cellular uptake. Entrapment efficiency (EE) of 4-HR was found to be 75.0—96.5%. The cytotoxicity of 4-HR-loaded SLNs demonstrated enhanced anti-cancer effects compared to pure 4-HR. The enhancement of anti-cancer effects depended on reduced particle size based on cellular uptake, the EE, and the cell type. Conclusions: These findings imply that 4-HR-loaded SLN is a promising strategy for chemotherapy in cancer treatment.

## 1. Introduction

Cancer is recognized as one of the most critical challenges to human health [1,2]. It is a life-threatening disease caused by unstoppable cell growth, leading to a progression of genomic alterations and their subsequent dissemination throughout the system [3,4,5]. Surgical removal, chemotherapy, and radiotherapy, either alone or in combination, are the key strategies for cancer treatment today [6]. Although surgical excision extensively removes malignant tumors as an initial approach, residual cancer cells can easily recur [7]. Chemotherapy, following surgical excision, proves to be an effective strategy against the remaining cancer cells due to its broad therapeutic effects on various cancers [7,8]. Drugs that have the effect of inducing cancer apoptosis or preventing the growth of cancer cells can be used as chemotherapy drugs [9,10,11].

4-hexylresorcinol (4-HR) emerges as a potential anti-cancer agent [9,10,11,12]. As a synthetic resorcinolic lipid, 4-HR has a wide array of pharmacological effects, including fighting microbes, reducing inflammation, and combating cancer [11,13,14]. With a molecular weight of 194.27 g/mol and a chemical name of 4-hexyl-1,3-phenylenediol, 4-HR exerts its anti-cancer effect by inhibiting histone deacetylase (HDAC). The action of HDACs hinders T cells from recognizing and destroying cancer cells [15,16,17]. Chemical analogs of 4-HR have shown anti-cancer effects in animal models of colon, lung, and pancreatic tumors [3,4,5]. However, the hydrophobic nature of 4-HR leads to aggregation, resulting in relatively poor bioavailability and pharmacological effects [18,19].

Conventional pharmaceutical strategies in cancer therapy entail cellular uptake associated with nano-sized particles (poly(lactic-co-glycolic) acid (PLGA) nanoparticles) [20] or lipid-based formulations (liposomes) [21]. Solid lipid nanoparticles (SLNs) are advantageous in cancer-targeted nanomedicines due to their lipid nature and nanoscale particle size [22,23,24,25,26]. In SLNs, a poorly water-soluble drug is stably encapsulated by fabricating an oil-in-water (O/W) phase via sonication [24,25,26,27]. Importantly, SLNs are prepared without using organic solvents, which reduces the risk of toxicity [28,29,30]. Drug aggregation can be avoided by encapsulating the drug into SLNs, which enhances tumor-targeted delivery.

The purpose of this study is to assess the enhancement of the anti-cancer effect of 4-HR encapsulated in SLNs. SLNs of 4-HR were prepared using sonication. The effects of the type and concentration of lipids and surfactants were assessed for particle size, polydispersity index (PDI), zeta potential, and drug release, as those factors affect the anti-cancer effect. The assessment of the anti-cancer effect involved three tumor cell lines, including HeLa (originating from human cervical cancer), A549 (from human lung carcinoma), and CT-26 (from mouse colon carcinoma).

## 2. Results and Discussion

### 2.1. Characterization of 4-HR

#### 2.1.1. Development of Analytical Method of 4-HR

Through the analysis of the absorption spectra and specificity of 4-HR, we determined the peak absorption wavelength and distinctive absorption characteristics, with the UV-Vis spectrum revealing a maximum absorption wavelength of 281 nm (Figure S1A). Analysis of the placebo SLN, which had the same composition as F1, revealed no interference between the placebo and the analyte. The analysis of 4-HR was conducted at 281 nm.

#### 2.1.2. Linearity

Figure S1B represents a calibration curve derived from the analysis of five 4-HR standard stock solutions diluted within the concentration range of 1–100 ppm. The calibration curve exhibited a correlation coefficient of 0.9964, as determined through linear regression analysis.

#### 2.1.3. Precision and Accuracy

Precision was assessed by measuring the proximity of values obtained from the same concentration of the standard stock solution [31,32]. This closeness was expressed as the relative standard deviation (RSD) of repeatability, which was found to be 1.1% for the recovery rate (Table S1). This result confirms the high precision of the proposed analysis method.

Accuracy was determined by evaluating the closeness between the obtained test result and the true or accepted reference value [31,32]. The RSD (%) values for drug recovery were determined to be 2.2%, 0.9%, and 2.5%, respectively (Table S2).

### 2.2. Nanoparticle Size, PDI, and Zeta Potential of 4-HR-Loaded SLNs

**Figure 1:**
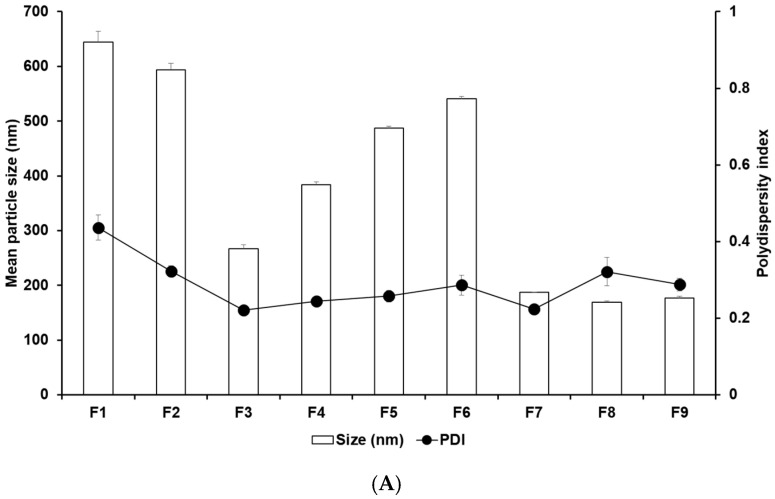

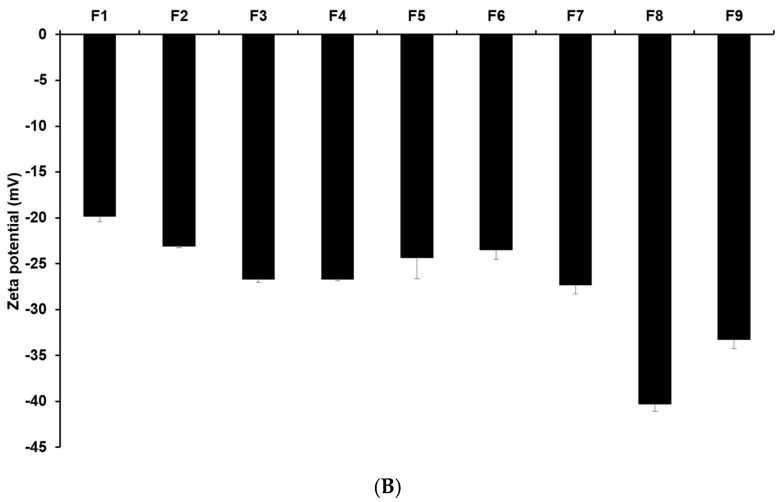
Nanoparticle characteristics of (**A**) particle sizes and PDIs and (**B**) zeta potentials of 4-HR-loaded SLNs prepared using different ingredients. Comparing the type of lipids, the type of surfactants, the concentration of surfactants, the concentration of 4-HR, and the concentration of lipids, particle size showed that SLNs using longer carbon chain fatty acids are small in size with higher zeta potential. Data are reported as the average ± standard deviation obtained from three independent trials (n = 3). PDI, polydispersity index.

Figure 1 and Tables S3 and S4 display the results of size, PDI, and zeta potential of SLNs with these confidence intervals. The efficacy of cancer therapy can be significantly affected by particle size, which is closely related to cellular uptake [22,23,33,34,35]. The average particle sizes of all formulations were 644.77 nm (F1), 594.03 nm (F2), 267.17 nm (F3), 383.80 nm (F4), 486.87 nm (F5), 540.57 nm (F6), 186.90 nm (F7), 169.43 nm (F8), and 176.63 nm (F9). By comparing the results of different lipids (F1, F2, F3, and F7), it was observed that F7 using glycerol monostearate (GMS) exhibited the smallest, most homogeneous, and most stable particles. This suggests that the hydrophobic property of 4-HR facilitates high dissolution into the lipid matrix of SLN [36,37,38]. Additionally, a lipid matrix using longer carbon chain fatty acids induces a greater affinity with 4-HR, resulting in a higher molecular dispersion of 4-HR in the lipid matrix [37,38]. The number of total carbon atoms in lauric acid (LAD), palmitic acid (PA), stearic acid (SA), and GMS is 12, 16, 18, and 21, respectively. Therefore, SLN formulated with GMS (F7) seems to be a suitable formulation for cellular uptake among the formulations using different lipids. The formulations using GMS (F7–F9) were under 200 nm, unlike F1–F6. This suggests that the effect of lipids of carbon chain lengths is dominant. The size results of F7 and F8 show that as the concentration of GMS decreases, the particle size decreases, and the stability of the particle increases. This indicates that the volume of the lipid matrix increases as the concentration of lipids increases [39,40]. Regarding the effect of the surfactant, the graph of F7 and F9 shows that the particle size was smaller and more stable as the amount of poloxamer 188 (PX 188) increased. PX 188 contributes to stabilizing the interface between the O phase and the W phase in the O/W emulsion [37,41,42,43]. In formulations using SA as lipids, F4 containing Tween 80 (TW 80) had a larger particle size than F3 containing PX 188. This indicates that PX 188, being more hydrophilic than TW 80, has an excellent ability to stabilize the interface [41,42]. The structure of TW 80 is sorbitan monooleate with polyethylene glycol 20, and the hydrophilic–lipophilic balance (HLB) value is 15. PX 188 is composed of polyoxyethylene (a) and polyoxypropylene (b) chains in the ratio a:b:a = 80:27:80, and the HLB value is 29. The high hydrophilicity (high HLB value) of hydrophilic surfactants prevents the aggregation of the O phase during O/W emulsion preparation, leading to a smaller particle size of the nanoparticles. Comparing the results of F3, F5, and F6, as the concentration of 4-HR decreased, the particle size became smaller and more uniform. In this regard, F3, with the lowest drug concentration, was the smallest and the most stable. When it comes to the effect of the amount of 4-HR, the graphs of F3, F5, and F6 demonstrate that an increase in the amount of 4-HR led to a larger particle size and lower stability. The reason for this is that the larger size is attributed to the high amount of 4-HR, and therefore the exposure of a negative charge on the particle surface decreases [37,38,39,44].

### 2.3. Determination of the Drug Loading Capacity

**Figure 2:**
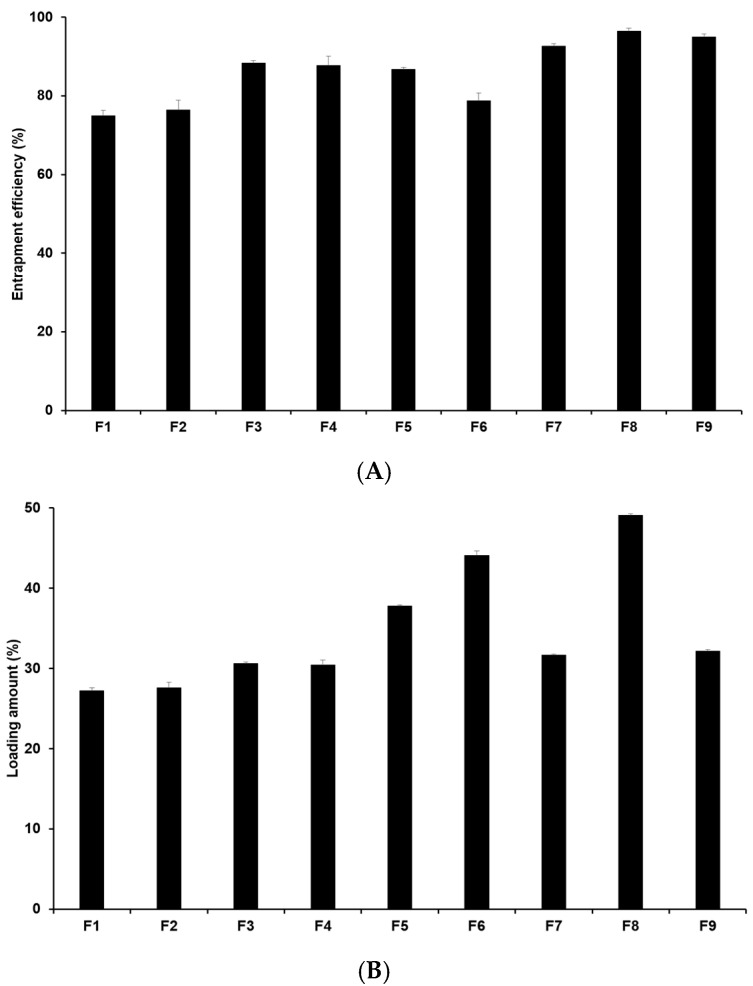
Drug loading capacity of 4-HR in SLNs. (**A**) Entrapment efficiency and (**B**) loading amount. It was observed that SLNs formulated with longer carbon chain fatty acids exhibited higher loading capacities when comparing the type of lipids, the type of surfactants, the concentration of surfactants, the concentration of 4-HR, and the concentration of lipids. The findings are expressed as mean values ± standard deviations based on three independent experiments (n = 3).

The drug loading capacity is crucial as high drug loading capacity is desirable for sustained drug release and for potentially reducing side effects by minimizing both the unencapsulated drug and the total drug dose required. Hence, it is an important parameter in drug delivery systems. Figure 2 illustrates that the entrapment efficiency (EE) of 4-HR-loaded SLNs ranges from 75.0% to 96.5%, with the loading amount (LA) ranging from 27.3% to 49.1% (Table S3). When comparing formulations using different lipids (F1, F2, F3, and F7), the loading capacities of 4-HR in SLNs increase with the carbon chain length of the lipid. In this sense, F7 containing GMS has the highest loading capacity among those formulations. This suggests that the strong affinity of 4-HR with lipids enhances the solubility of 4-HR in the lipid matrix, as mentioned regarding particle size [37,38]. Regarding the effect of surfactant, F7-F9 in Figure 2 shows that the loading capacity of 4-HR in SLNs increases with higher surfactant concentrations. This increased loading capacity is caused by the stabilization of the interface between the O and W phases [41,42]. In the case of F3 and F4, the EE and LA of 4-HR in F3 were slightly higher than those in F4. This suggests that PX 188 (F3) exhibits higher hydrophilicity than TW 80 (F4). A relatively high hydrophilic surfactant is capable of stabilizing the interface between the O and W phases, which results in a greater amount of 4-HR encapsulated in the SLN matrix [41,43].

### 2.4. In Vitro Drug Release Study

**Figure 3:**
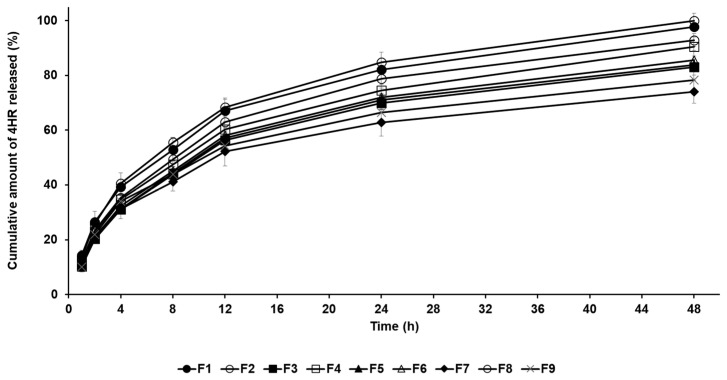
Cumulative percentage release profiles of 4-HR from SLNs F1—F9 in release medium. The observed order of 4-HR release rates suggests delayed release with increasing carbon chain length of the lipid when comparing the type of lipids, the type of surfactants, the concentration of surfactants, the concentration of 4-HR, and the concentration of lipids. Data are reported as the average ± standard deviation obtained from three independent trials (n = 3).

The drug release profile of 4-HR-loaded SLNs was conducted by the dialysis membrane method. In Figure 3, all formulations exhibited sustained release behavior for 48 h, which is attributed to the entrapment of the drug within the lipid matrix of the nanoparticles [24,25,26,27]. The amount order of 4-HR release from SLNs after 48 h was F8 (100.01%) > F1 (97.74%) > F2 (92.87%) > F4 (90.48%) > F6 (85.63%) > F5 (83.90%) > F3 (83.10%) > F9 (78.32%) > F7 (74.02%). When comparing the drug release rates of formulations using different lipids, the order was F1 > F2 > F3 > F7. This order indicates a delayed release as the carbon chain length of the lipid increases. This means that SLNs containing lipids with a strong affinity for 4-HR tend to inhibit drug release, as mentioned in particle size and loading capacity [36,37,38]. The graphs of F7 and F8 show that drug release is delayed when the lipid concentration increases. The drug release rate of F8 with lower lipid concentration was the fastest among all formulations. The reason for this is that the inhibitory effect on drug migration by the concentration of lipids is relieved, as previously mentioned [45]. Regarding surfactants (F3 and F4), the drug release of F3 using PX 188 was suppressed compared to F4 using TW 80. This suggests that a relatively stable formulation using PX 188 affects the inhibition of drug migration, resulting in delayed drug release [41,42].

### 2.5. In Vitro Cytotoxicity Evaluation

**Figure 4:**
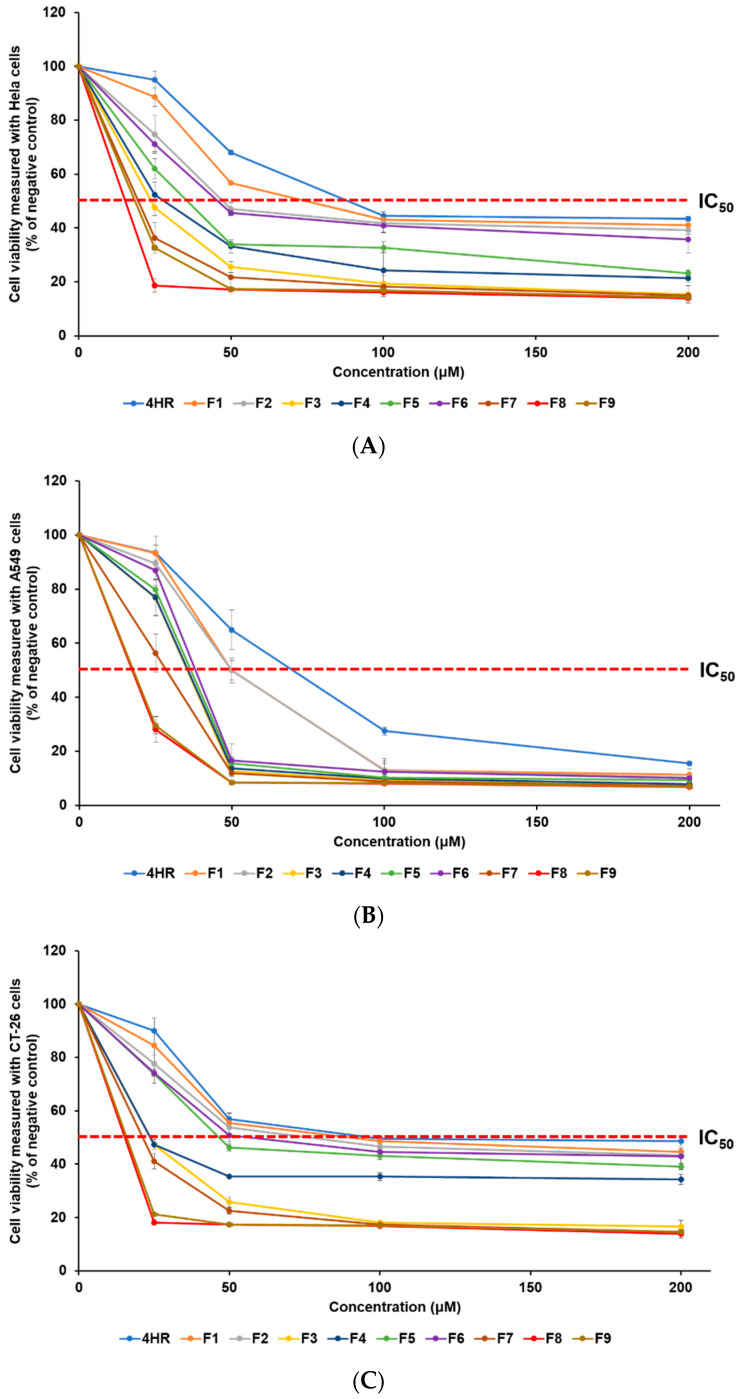
The WST assay was employed to measure the viability of three cancer cell lines, (**A**) HeLa, (**B**) A549, and (**C**) CT-26, treated with a pure 4-HR solution and SLNs F1—F9. The anti-cancer efficacy of 4-HR was enhanced by employing SLNs that facilitated the cellular uptake of 4-HR. The findings are expressed as mean values ± standard deviations based on three separate experiments (n = 3).

The anti-cancer effect of 4-HR on various cancer cell lines was assessed using the WST assay. This research involved the use of HeLa, A549, and CT-26 cell lines, representing human cervical cancer, human lung epithelial cancer, and mouse colorectal cancer, respectively. The anti-cancer effect of 4-HR-loaded SLNs was determined from the viability of cancer cells. To estimate the inhibitory medium concentration (IC_50_) values, samples were incubated at concentrations of 0, 25, 50, 100, and 200 μM, as detailed in Table 1. Figure 4 illustrates the analysis of cancer cell viability after treatment, revealing that all formulations exhibit an increase in anti-cancer effects in a concentration-dependent manner. The order of anti-cancer efficacy was identical in the three cell lines, according to the IC_50_ findings: F8 > F9 > F7 > F3 > F4 > F5 > F6 > F2 > F1 > 4HR. The obtained IC_50_ results of 4-HR were 88.5 μM (HeLa cells), 70.0 μM (A549 cells), and 97.2 μM (CT-26 cells). Although the anti-cancer mechanism of 4-HR has not been clearly revealed for all cancer cell lines, previous reports have suggested possible anti-cancer mechanisms, including hindering HDACs, the NF-kB pathway, transglutaminase, and calcium oscillations [9,10,11,15,16,17]. The cell type-dependent anti-cancer effect of 4-HR may be due to different anti-cancer mechanisms. In our previous report, curcumin, as a chemotherapy drug, showed a cell type-dependent anti-cancer effect depending on the different anti-cancer mechanisms of each cancer cell [32]. All SLNs exhibited IC_50_ values that were lower than the IC_50_ of pure 4-HR, indicating an improved anti-cancer effect for all formulations, compared to pure 4-HR. F8, which showed the best anti-cancer effect among SLNs, exhibited an enhanced anti-cancer effect of approximately 5.7 times (HeLa), 4.0 times (A549), and 6.4 times (CT-26), compared to pure 4-HR. This suggests that the lipid nature and the nano-sized particle of SLN influence the cellular uptake of 4-HR in cancer cells. Consequently, more 4-HR is delivered into the cancer cells, resulting in an enhanced anti-cancer effect [1,22,23,33,35]. The effect of differences in lipids used in the formulations and GMS concentrations was assessed through cytotoxicity analysis. When comparing F1, F2, F3, and F7, formulations using lipids with longer carbon chain lengths exhibited relatively higher anti-cancer effects related to particle sizes [33,34,35,37,38]. Particularly, F7 containing GMS exhibited a particle size over three times smaller than F1 using LAD, resulting in a significantly higher anti-cancer effect. From these results, it is inferred that the type of lipid influences both particle size and anti-cancer effects [33,35]. When it comes to the effect of GMS concentration, F8, with a lower GMS concentration, had the greatest anti-cancer effect.

## 3. Materials and Methods

### 3.1. Materials

4-HR was a gift from Barunbarum Co. (Seoul, Republic of Korea). We purchased SA, PA, and LAD from Samchun Co. (Pyeongtaek, Republic of Korea); GMS from Kanto Chemical Co., Japan. Inc. (Tokyo, Japan); PX 188 from BASF Co. Ltd. (Ludwigshafen, Germany); and TW 80 and sodium lauryl sulfate (SLS) from Dae Jung Co. Ltd. (Busan, Republic of Korea). PBS (phosphate-buffered saline) was obtained from Sigma-Aldrich Co. (St. Louis, MO, USA). Cancer cell lines (HeLa, A549, CT-26) were acquired from the Korea Cell Line Bank (Seoul, Republic of Korea). The Quanti-MAX WST-8 assay kit was provided by Biomax (Seoul, Republic of Korea). HPLC-grade methanol (MeOH) was purchased from Honeywell (Seelze, Germany). All other chemicals were also HPLC grade.

### 3.2. Preparation of 4-HR-Loaded SLNs

SLNs containing 4-HR were prepared using a modified O/W emulsion method via sonication [31]. Table 2 shows the various compositions of 4-HR-loaded SLNs. The O phase was prepared by dissolving 50—100 mg of 4-HR into 50—100 mg of the melted lipid at 10 °C above the lipid melting point. The well-known melting points of lipids in the literature are 44.8 °C (LAD), 62.9 °C (PA), 70.1 °C (SA), and 78–81 °C (GMS). Using a homogenizer (PT 3100; Kinematica Instruments, Luzerne, Switzerland), the mixture was homogenized at a speed of 5000 rpm. The W phase, dissolved with 200–400 mg of the surfactant, was added to the O phase, followed by homogenization at 1000 rpm. For nanonization, the obtained O/W emulsion was treated with a probe sonicator (Scientz-IID, Ningbo, China) at 300 W for 15 min, with a cycle consisting of 5 s on and 5 s off.

### 3.3. Characterization of 4-HR

#### 3.3.1. Development of Analytical Method for 4-HR

The UV-Vis spectrophotometer (S-3100, Scinco, Seoul, Republic of Korea) was employed to analyze the concentration of 4-HR at room temperature [31]. The wavelength of maximum absorption for 4-HR was determined within the range of 250 to 800 nm. A standard stock solution was made by completely dissolving 2 mg of 4-HR in 20 mL of MeOH.

#### 3.3.2. Linearity

Linearity was evaluated from the UV-Vis absorption spectra after serially diluting the completely dissolved standard solution with MeOH to prepare 5 concentrations. Concentration versus absorbance units and calibration curves were prepared for the measurement of 4-HR.

#### 3.3.3. Precision and Accuracy

Precision and accuracy were analyzed by the UV-Vis absorption spectra. Precision was evaluated by measuring the stock solution six times and calculating the RSD of the assay results. To evaluate the accuracy, the recovery rate of the stock solution was set to 0%, 25%, and 100%, and the relative RSD was also calculated.

### 3.4. Measurements of Nanoparticle Size, PDI, and Zeta Potential of 4-HR-Loaded SLNs

Measurements of particle size, PDI, and zeta potential for the SLNs were conducted at 25 °C using dynamic light scattering and electrophoretic light scattering techniques with a Zetasizer Nano ZS (Malvern Instruments Ltd., Worcestershire, Malvern, UK) [46]. Samples were prepared by diluting them tenfold with distilled water (DW) and were measured three times to obtain the average. Each measurement was performed in triplicate.

### 3.5. Determination of Drug Loading Capacity

To analyze the drug loading capacity, the 4-HR-loaded SLNs were diluted and centrifuged at 1300 rpm at 4 °C for 1 h [46]. The concentration of free drug in the supernatant was measured using UV-Vis, as described in Section 2.3. The EE and LA were calculated using the following equations in triplicate:

$$EE\;\left(\%\right)=\frac{Amount\;of\;total\;drug\;content\;-\;Amount\;of\;free\;drug}{Amount\;of\;total\;drug\;content}\times 100$$

$$LA\;\left(\%\right)=\frac{Amount\;of\;total\;drug\;content\;-\;Amount\;of\;free\;drug}{\left(Amount\;of\;total\;drug\;content\;-\;Amount\;of\;free\;drug\right)+Amount\;of\;lipid}\times 100$$

### 3.6. In Vitro Drug Release Studies

The in vitro release of 4-HR was assayed using the dialysis bag method [31,46]. The test substance in dialysis bags (Spectrum Laboratories, Inc., Compton, CA, USA, molecular weight: 10 kDa) was immersed in vials containing 50 mL of PBS with 1% of SLS. The vials were shaken in a shaking incubator set at 100 rpm and 37 ± 0.5 °C. After 1, 2, 4, 8, 12, 24, and 48 h, 1 mL of the medium was withdrawn. All samples in triplicate were immediately analyzed using UV-Vis, as described in Section 2.3.

### 3.7. In Vitro Cytotoxicity Evaluation

#### 3.7.1. Cytotoxicity Study Using Tumor Cell Lines

The efficacy of 4-HR-loaded SLNs in combating cancer was evaluated by testing their cytotoxicity on three different tumor cell lines: HeLa (human cervical carcinoma), A549 (human lung carcinoma), and CT-26 (mouse colon carcinoma) [31,46]. Cells were plated into 48-well plates at 2 × 10^4^ cells per well and counted with a hemocytometer. After a 24 h incubation period at 37 ± 0.5 °C and 5% CO_2_, the cells were treated with various concentrations of the samples (1, 2.5, 5, and 10 μM). Following another 24 h, the cells were washed with sterile PBS, and 200 μL of growth medium was added. Following this, the cells were incubated for an additional 24 h at 37 ± 0.5 °C with 5% CO_2_ before the WST reduction assays were conducted.

#### 3.7.2. Viability of Cancer Cells

Cell viability was conducted by incubating cells in a 48-well plate with a 10% WST solution for 1 h. The optical density (OD) of WST that reacted with viable cells was measured using a microplate reader at 450 nm. Once the blank OD was subtracted from the raw data, the mean OD value ± standard deviation (SD) was obtained from three replicates per test substance. Cell viability was calculated using the following equation, with the negative control (NC) value set to 100%:

$$Viability\;\left(\%\right)=\frac{{Mean\;OD}_{treated}}{{Mean\;OD}_{control}}\times 100$$

## 4. Conclusions

The purpose of this study is to design and develop SLNs loaded with 4-HR for use in chemotherapy-based anti-cancer treatments. The different characteristics of each SLN were evaluated according to the type and amount of lipids and surfactants. SLN formulations containing GMS had the smallest, most homogeneous, and most stable particles compared to those using LAD, PA, and SA. In addition, formulations containing GMS had the highest EE and LA. The loading capacity of 4-HR tended to increase with higher concentrations of lipids and surfactants. As a result of analyzing the drug release profile of 4-HR-loaded SLNs, formulations using GMS revealed sustained drug release rates for the longest duration. Conversely, formulations containing lipids with relatively shorter carbon chain lengths or lower lipid concentrations showed relatively rapid drug release rates. Cytotoxicity was evaluated using three tumor cell lines (HeLa, A549, and CT-26). Although the anti-cancer mechanism of 4-HR has not yet been identified, the cell type-dependent anti-cancer effect of 4-HR was shown. In all formulations, the anti-cancer effect of 4-HR was enhanced by loading SLNs in a particle size-dependent manner. In this regard, this suggests that the lipid nature and nanoparticle size of SLNs facilitate the cellular uptake of 4-HR. The results of this study indicate that 4-HR-loaded SLNs hold promise as an effective approach for anti-cancer chemotherapy.

## Figures and Tables

**Table 1:** IC_50_ (μM) value of HeLa, A549, and CT-26 cells; particle size and entrapment efficiency (EE) of pure 4-HR solution, F1—F9.

	Hela (μM)	A549 (μM)	CT-56 (μM)	Particle Size (nm)	EE (%)
4-HR	88.5	70.0	97.2	N/A	N/A
F1	74.5	50.0	90.3	644.8 ± 19.3	75.0 ± 1.3
F2	47.4	49.9	76.4	594.0 ± 11.1	76.4 ± 2.5
F3	23.8	35.5	23.8	267.2 ± 6.6	88.4 ± 0.6
F4	28.0	35.7	23.8	383.8 ± 4.9	87.7 ± 2.4
F5	35.8	36.6	46.6	486.9 ± 3.6	86.8 ± 0.4
F6	45.7	38.1	56.7	540.6 ± 4.3	78.8 ± 1.9
F7	19.6	28.6	21.2	186.9 ± 1.2	92.6 ± 0.6
F8	15.4	17.4	15.3	169.4 ± 2.5	96.5 ± 0.7
F9	18.6	17.8	15.9	176.6 ± 3.7	95.0 ± 0.8

N/A, not applicable.

**Table 2:** Composition of 4-HR-loaded SLNs.

Formulation		F1	F2	F3	F4	F5	F6	F7	F8	F9
Drug (mg)	4-HR	50	50	50	50	70	100	50	50	50
Lipid (mg)	LAD	100								
	PA		100							
	SA			100	100	100	100			
	GMS							100	50	100
Surfactant (mg)	PX 188	200	200	200		200	200	200	200	400
	TW 80				200					

4-HR, 4-hexylresorcinol; LAD, lauric acid; PA, palmitic acid; SA, stearic acid; GMS, glycerol monostearate; PX 188, poloxamer 188; TW 80, Tween 80.

## Data Availability

The data presented in this study are available in [4-hexylresorcinol-loaded solid lipid nanoparticles for enhanced cancer chemotherapy].

## Supplementary Materials

The following supporting information can be downloaded at: https://www.mdpi.com/article/10.3390/ph17101296/s1, Figure S1. UV-Vis spectra and a calibration curve of 4-HR. The maximum absorption wavelength of 4-HR was 281 nm. (A) Specificity data for 4-HR, placebo, and 4-HR-loaded SLN F1 (MeOH, 25 °C). (B) Linearity data of 4-HR standard stock solution in MeOH; Table S1. Precision data of 4-HR was obtained from the developed analytical method; Table S2. Accuracy data of 4-HR was obtained from the developed analytical method; Table S3. Nano-particle characteristics of particle sizes, polydispersity indexs (PDIs), zeta potentials, entrapment efficiency, and loading amount of 4-HR-loaded SLNs; Table S4. Confidence interval of nano-particle characteristics for 4-HR-loaded SLNs;
