# Supplementary material for: 4-Hexylresorcinol Loaded Solid Lipid Nanoparticles for Enhancing Anticancer Activity
Source: Pharmaceuticals (Basel). 2024 Sep 29;17(10):1296. doi: 10.3390/ph17101296 (PMC11514591; doi:10.3390/ph17101296)
Supplement: Supplementary file 1 [file pharmaceuticals-17-01296-s001.zip › pharmaceuticals-3193245-supplementary.pdf]

Supporting Information

4-Hexylresorcinol Loaded Solid Lipid Nanoparticles for Enhancing Anticancer Activity

Sooho Yeo 1,*,† , Sukkyun Jung 2,†, Haneul Kim 1, Jun-Hyun Ahn 3 and Sung-Joo Hwang 1,*

[1] *Yonsei Institute of Pharmaceutical Sciences, College of Pharmacy, Yonsei University, Incheon 21983, Republic of Korea; 1543sky@naver.com*

[2] *Research Center of Barunbarum Co., Seoul 06776, Republic of Korea; jsk0314@barunbarum.com*

[3] *Department of Biopharmaceutical Engineering, Hannam University, 1646 Yuseongdae-ro, Yuseong-gu, Daejeon 34054, Republic of Korea; ajh@hnu.kr*

* Correspondence: sooho32@yonsei.ac.kr (S.Y.); sjh11@yonsei.ac.kr (S.-J.H.);

Tel.: +82-32-749-4173 (S.Y.); +82-32-749-4518 (S.-J.H.)

† These authors contributed equally to this work.

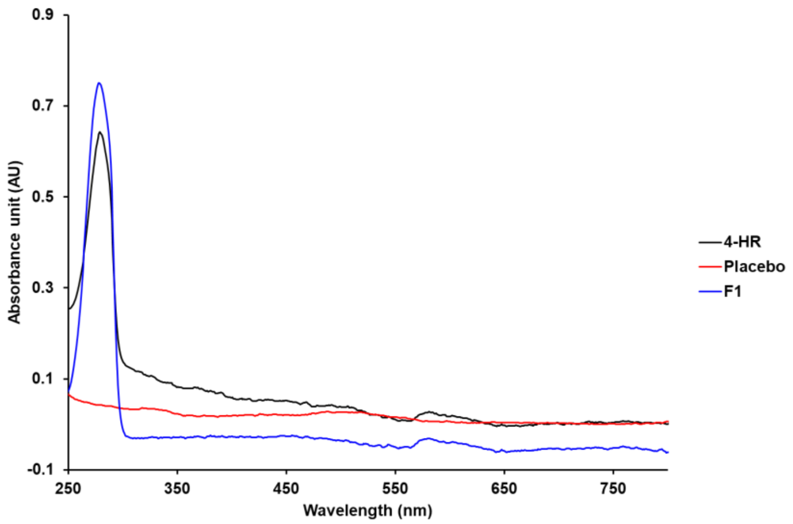

(**A**)

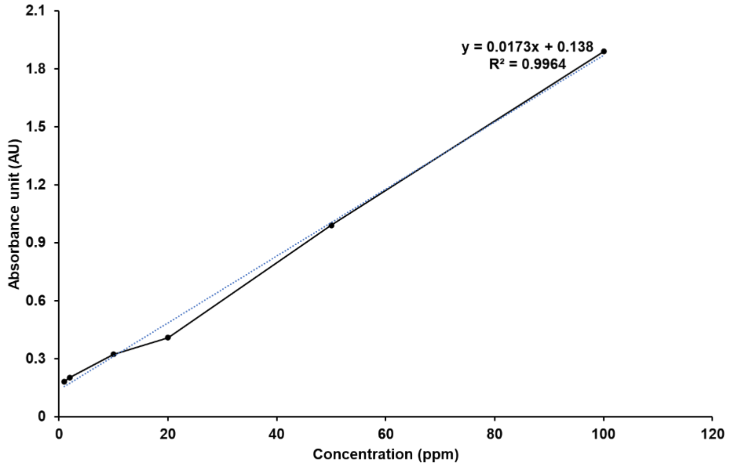

(**B**)

Figure S1. UV-Vis spectra and a calibration curve of 4-HR. The maximum absorption wavelength of 4-HR was 281 nm. (A) Specificity data for 4-HR, placebo, and 4-HR-loaded SLN F1 (MeOH, 25 °C). (B) Linearity data of 4-HR standard stock solution in MeOH.

Table S1. Precision data of 4-HR was obtained from the developed analytical method.

No	Recovery (%)
1	98.3
2	100.0
3	98.1
4	96.9
5	96.5
6	98.0
Average	98.0
SD	1.1
RSD (%)	1.1

Table S2. Accuracy data of 4-HR was obtained from the developed analytical method.

Drug (ppm)	No.	Recovery (%)	Average	SD	RSD (%)
	1	105.0			
1	2	100.0	103.2	2.3	2.2
	3	104.6			
	1	98.3			
10	2	100.0	98.8	0.9	0.9
	3	98.1			
	1	106.1			
100	2	100.0	102.6	2.6	2.5
	3	101.5			

Table S3. Nano-particle characteristics of particle sizes, polydispersity indexs (PDIs), zeta potentials, entrapment efficiency, and loading amount of 4-HR-loaded SLNs

	Size (nm)	PDI	Zeta potential (mV)	Entrapment efficiency (%)	Loading amount (%)
F1	644.77 ± 19.33	0.44 ± 0.03	-19.83 ± 0.62	74.95 ± 1.31	27.26 ± 0.35
F2	594.03 ± 11.14	0.32 ± 0.01	-23.07 ± 0.21	76.37 ± 2.53	27.63 ± 0.66
F3	267.17 ± 6.60	0.22 ± 0.01	-26.70 ± 0.36	88.40 ± 0.62	30.65 ± 0.15
F4	383.80 ± 4.92	0.24 ± 0.00	-26.67 ± 0.21	87.70 ± 2.40	30.48 ± 0.58
F5	486.87 ± 3.55	0.26 ± 0.01	-24.33 ± 2.30	86.80 ± 0.41	37.80 ± 0.11
F6	540.57 ± 4.26	0.29 ± 0.03	-23.50 ± 1.02	78.82 ± 1.91	44.07 ± 0.60
F7	186.90 ± 1.20	0.22 ± 0.01	-27.33 ± 0.95	92.64 ± 0.60	31.66 ± 0.14
F8	169.43 ± 2.54	0.32 ± 0.04	-40.30 ± 0.80	96.48 ± 0.73	49.10 ± 0.19
F9	176.63 ± 3.65	0.29 ± 0.02	-33.23 ± 1.01	95.03 ± 0.77	32.21 ± 0.18

Table S4. Confidence interval of nano-particle characteristics for 4-HR-loaded SLNs

	Size			PDI			Zeta potential		
	90%	95%	99%	90%	95%	99%	90%	95%	99%
F1	[626, 663]	[623, 667]	[616, 673]	[0.405, 0.468]	[0.399, 0.474]	[0.387, 0.486]	[-20.4, -19.2]	[-20.5, -19.1]	[-20.8, -18.9]
F2	[583, 605]	[581, 607]	[577, 611]	[0.308, 0.336]	[0.306, 0.339]	[0.3, 0.344]	[-23.3, -22.9]	[-23.3, -22.8]	[-23.4, -22.8]
F3	[261, 273]	[260, 275]	[257, 277]	[0.213, 0.228]	[0.212, 0.23]	[0.209, 0.233	[-27, -26.4]	[-27.1, -26.3]	[-27.2, -26.2]
F4	[379, 388]	[378, 389]	[376, 391]	[0.24, 0.248]	[0.24, 0.249]	[0.238, 0.25]	[-26.9, -26.5]	[-26.9, -26.4]	[-27, -26.4]
F5	[484, 490]	[483, 491]	[482, 492]	[0.244, 0.272]	[0.241, 0.274]	[0.236, 0.28]	[-26.5, -22.1]	[-26.9, -21.7]	[-27.8, -20.9]
F6	[537, 545]	[536, 545]	[534, 547]	[0.262, 0.311]	[0.257, 0.315]	[0.248, 0.324]	[-24.5, -22.5]	[-24.6, -22.4]	[-25, -22]
F7	[186, 188]	[186, 188]	[185, 189]	[0.213, 0.234]	[0.211, 0.236]	[0.207, 0.24]	[-28.2, -26.4]	[-28.4, -26.3]	[-28.7, -25.9]
F8	[167, 172]	[167, 172]	[166, 173]	[0.287, 0.356]	[0.28, 0.363]	[0.267, 0.376]	[-41.1, -39.5]	[-41.2, -39.4]	[-41.5, -39.1]
F9	[173, 180]	[173, 181]	[171, 182]	[0.272, 0.303]	[0.269, 0.306]	[0.263, 0.312]	[-34.2, -32.3]	[-34.4, -32.1]	[-34.7, -31.7]
